# Quadratus lumborum block for postoperative pain management in patients undergoing ileostomy closure: a prospective, randomized controlled trial

**DOI:** 10.12701/jyms.2026.43.5

**Published:** 2025-12-19

**Authors:** Su Jin Kang, Soo Yeun Park, Jun Seok Park, Jinseok Yeo

**Affiliations:** 1 Colorectal Cancer Center, Kyungpook National University Chilgok Hospital, Daegu, Korea; 2 Department of Anesthesiology and Pain Medicine, Kyungpook National University Chilgok Hospital, Daegu, Korea

**Keywords:** Analgesia, Ileostomy, Nerve block, Postoperative pain

## Abstract

**Background:**

Quadratus lumborum (QL) block is used for multimodal analgesia following abdominal surgery. We introduced an ultrasound-guided QL block to treat postoperative pain for ileostomy closure. This study aimed to investigate the analgesic efficacy of the QL block compared to placebo after ileostomy closure.

**Methods:**

Fifty-seven patients undergoing elective ileostomy closure were randomized (1:1) to the placebo or QL block group in this double-blind randomized controlled trial. After general anesthesia, a unilateral QL block was performed under ultrasound guidance. Opioid consumption and numeric rating scale (NRS, 0–10) pain scores were recorded at 2, 6, 12, 24, 48, and 72 hours postoperatively. The primary outcome was the NRS pain score at rest at 6 hours. Secondary outcomes included pain scores, rescue analgesics over 72 hours, Quality of Recovery-15 scores in 24 hours, complications, and length of hospital stay.

**Results:**

Baseline characteristics were similar among the 54 patients (27 per group) who completed the study, excluding three who dropped out. The QL block did not reduce NRS pain scores at rest at 6 hours (median [interquartile range], 5 [4–6] vs. 5 [3–6]; *p*=0.78). Over the 72-hour postoperative period, pain scores at rest remained comparable between the groups, while the QL group showed slightly lower movement-induced pain at certain time points. The QL group required fewer analgesics and antiemetics at certain intervals, but the total opioid use, length of hospital stay, and quality of recovery were not significantly different.

**Conclusion:**

The QL block showed no meaningful advantage in postoperative analgesia compared to placebo for ileostomy closures.

## Introduction

As surgical methods evolve toward minimally invasive techniques, the number of studies focusing on pain control at the incision site after abdominal surgery has increased. Postoperative pain is not only a source of suffering for patients but is also associated with several life-threatening complications such as atelectasis, pneumonia, hypoxemia, hypertension, and deep vein thrombosis, along with issues such as nausea, ileus, and anxiety [1,2]. Research on multimodal analgesia to reduce postoperative pain is ongoing, and several medical centers commonly use opioid analgesics and patient-controlled analgesia devices. However, opioids used for pain control are associated with side effects including nausea, vomiting, respiratory depression, urinary retention, and pruritus, which can discourage patients from using them [3,4]. Consequently, local and regional anesthetics are gaining attention to minimize the adverse effects of systemic opioids [5].

Recent studies have proposed the use of local and regional anesthetics, such as the transversus abdominis plane block [6-8] or local infiltration [9], at the incision site in patients undergoing abdominal surgery. These methods involve blocking the nerves that innervate the surgical site and can be used in surgeries performed under local anesthesia as well as at the incision sites of surgeries conducted under general anesthesia. These approaches are increasingly integrated into pain management protocols because of their effectiveness in reducing opioid use and alleviating patient discomfort [10,11].

The quadratus lumborum (QL) block, first proposed by Blanco in 2007, involves the infiltration of a local anesthetic agent adjacent to the anterolateral aspect of the QL muscle and has since been further studied and developed [12,13]. The somatic nerves from T6 to L1, and some sympathetic nerves associated with abdominal pain, can be effectively blocked using a QL block. The QL block, which involves injecting an anesthetic near the spinal nerves, offers longer-lasting pain relief and is more effective than the transverse abdominis plane (TAP) block [14]. QL block has been observed to effectively reduce postoperative pain in cesarean sections and laparoscopic abdominal or gynecological surgeries [15,16].

In rectal surgeries, the rate of temporary ileostomy construction to protect the colorectal anastomosis varies considerably, ranging from 3.9% to 62.5%, and is influenced by patient- and surgery-related risk factors [17]. Temporary ileostomy is typically reversed through secondary surgery, usually performed more than 2 months after the initial rectal resection. Unilateral administration of a QL block is feasible because the ileostomy reversal is confined to the right lower abdomen. To the best of our knowledge, the efficacy of the QL block in ileostomy reversal surgery has not been reported in any prospective studies to date. This study aimed to validate the hypothesis that the QL block is effective for pain control by conducting a randomized, double-blind study comparing a placebo group with a ropivacaine group.

## Methods

**Ethics statement:** This study was approved by the Ethics Committee of Kyungpook National University Chilgok Hospital (approval number: KNUCH 2017-12-011). Written informed consent was obtained from all patients prior to the surgery, in accordance with the principles of the Declaration of Helsinki. The study report adhered to the guidelines of the Consolidated Standards of Reporting Trials.

### 1. Trial design

This trial was conducted at the Kyungpook National University Chilgok Hospital (Daegu, Republic of Korea) between April 2020 and March 2024. It was designed as a parallel trial with equal allocations to compare the QL and placebo groups. This study aimed to demonstrate the superiority of QL block for pain management after ileostomy reversal. This study was registered at ClinicalTrials.gov (https://clinicaltrials.gov/study/NCT03411096).

### 2. Participants

Patients aged 20 to 75 years and scheduled for ileostomy reversal surgery, with an American Society of Anesthesiologists (ASA) physical status classification of I to II and normal liver and renal functions, were eligible to participate in the study. Exclusion criteria included morphine allergy, drug dependence, chronic pain, sleep apnea, asthma, chronic obstructive pulmonary disease, psychiatric disorders, uncontrolled diabetes mellitus, history of vascular interventions due to heart disease, bleeding tendencies, or potential pregnancy. Patients with asthma or chronic obstructive pulmonary disease were excluded because of an increased risk of postoperative pulmonary complications and potential bias in symptom assessment [18,19]. Patients who had received analgesics within 24 hours prior to surgery were also excluded from the study.

### 3. Randomization and blinding

Patients were enrolled after obtaining preoperative consent for the study. On the day of surgery, patients were assigned to either the control or intervention group in a 1:1 ratio by applying computerized randomization with block sizes of 2 and 4 in equal proportions. The randomization information was conveyed to the operating room by clinical research personnel. An operating room nurse prepared 20 mL of 0.25% ropivacaine or an equal volume of normal saline using an unlabeled syringe. The participants were not informed of the assigned group. An independent clinical researcher blinded to the group assignments performed outcome measurements.

### 4. Intervention

**Fig. 1.**
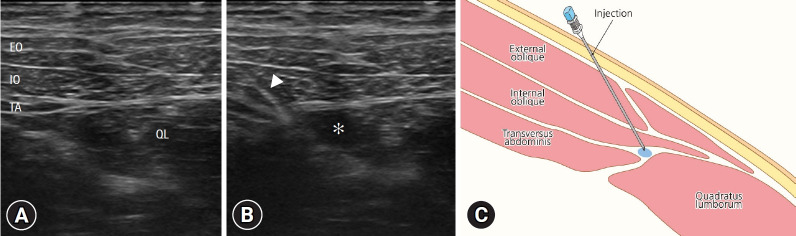
(A) Pre-injection and (B) post-injection ultrasound-guided images of quadratus lumborum (QL) blocks. ▲, needle; ✻, the spread of local anesthetics in quadratus lumborum. (C) Schematic representation of the QL block technique. EO, external oblique; IO, internal oblique; TA, transversus abdominis.

Standard monitoring was performed upon patient arrival at the operating room. Anesthesia was induced with propofol (1.5 mg/kg) and rocuronium (0.4 mg/kg), and maintenance was achieved with desflurane (4%–8%) in a 50% oxygen-air mixture and with remifentanil infusion (2–4 ng/mL). Rocuronium was administered for muscle relaxation and Hartmann solution was infused at 6 to 12 mL/kg/hour. No supplemental analgesics were administered. The QL block was performed unilaterally under ultrasound guidance with the patient placed in the prone position after the administration of general anesthesia by an experienced anesthesiologist. After identifying the right QL muscle, the drug was injected at the anterolateral border of the muscle and at its junction with the transversalis fascia (Fig. 1). The placebo group received 20 mL of saline, whereas the QL group received 20 mL of 0.25% ropivacaine. Four surgeons performed the ileostomy closure. After a small incision was made along the ileostomy, the surgeon carefully dissected the surrounding tissue to separate the ileostomy from the abdominal wall. Hand-sewn sutures or intestinal staples were used to close the bowel opening. Abdominal fascia closure was performed with continuous or interrupted sutures using 1-0 Polysorb (Medtronic, Mansfield, MA, USA), followed by skin closure with a vertical mattress suture using 3-0 nylon or a purse-string suture using 3-0 Vicryl (Ethicon, Somerville, NJ, USA) and 2-0 nylon.

**Fig. 2.**
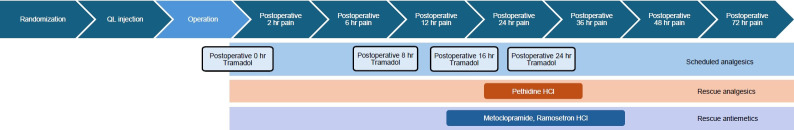
Flowchart of procedures and assessment time points, including postoperative scheduled analgesic administration and the time points for rescue analgesic or antiemetic use. QL, quadratus lumborum; HCl, hydrochloride.

After surgery, tramadol (50 mg) mixed with 100 mL of normal saline was intravenously administered in the ward at 8-hour intervals during the first 24 hours. Thereafter, tramadol was prescribed on-demand at a minimum interval of 4 hours, with a maximum daily limit of 400 mg. Patients who did not achieve adequate analgesia with tramadol, even after a minimum 1-hour response assessment, were administered pethidine (25 mg) mixed with 100 mL of normal saline as a rescue analgesic, with a maximum daily limit of 600 mg. Histamine-2 receptor antagonists were administered twice daily during the fasting period until postoperative day 3. Routine antiemetics were not administered; instead, metoclopramide or ramosetron hydrochloride was administered on demand (Fig. 2).

### 5. Outcomes

At each time point, each patient’s pain was assessed using a survey, and the administration of rescue analgesics and antiemetics prescribed upon patient request were recorded. The primary outcome was pain at rest reported 6 hours after surgery, quantified based on the numeric rating scale (NRS; 0, no pain; 10, worst pain imaginable). In this study, NRS at postoperative 6 hours was chosen as the primary outcome to evaluate the efficacy of preemptive QL block in alleviating patient-reported postoperative pain beyond the half-life of ropivacaine. The secondary outcomes included the NRS pain score at rest at 2, 12, 24, 36, 48, and 72 hours postoperatively; movement-induced pain at 2, 6, 12, 24, 36, 48, and 72 hours postoperatively; number of patients who received additional analgesics beyond those scheduled; number of patients who had postoperative nausea and vomiting (PONV) up to 72 hours; frequency of postoperative metoclopramide or ramosetron HCl administration; amount of intraoperative fluids and anesthetic agents administered; and duration of general anesthesia and surgery. Additional outcomes included post-anesthesia care unit (PACU) time, Quality of Recovery-15 (QoR-15) scale at 24 hours after surgery, time to first flatus, incision length, length of hospital stay, 30-day postoperative complications as graded by the Clavien–Dindo classification [20], and postoperative readmission.

### 6. Sample size calculation

The sample size was calculated based on a pilot study that included 25 patients who had undergone ileostomy closure and received a scheduled tramadol injection for 24 hours postoperatively. The mean NRS pain score at rest at postoperative 6 hours was 7.1 with a standard deviation of 1.1. Assuming a reduction in this pain score by 1 after a significant QL block, a total of 27 patients per group was estimated to yield 95% power at two-tailed α=0.05, and a requirement of 30 patients per group was estimated considering 10% follow-up loss. PASS ver. 14.0.8 (power analysis and sample size software; NCSS, Kaysville, UT, USA) was used to calculate the number of cases required.

### 7. Statistical analysis

All statistical analyses, including all randomized patients, were conducted according to intention-to-treat analysis. Categorical variables are presented as numbers (%). The chi-square test or Fisher exact test was used to compare two groups. Continuous variables were compared using a t-test or Mann–Whitney U-test and expressed as means with standard deviations or as medians with interquartile ranges (IQRs). Equality of variance with the Levene test was performed for continuous variables, and the normal distribution of the data was confirmed by limited skewness and kurtosis. IBM SPSS ver. 27.0 (IBM Corp., Armonk, NY, USA) was used for statistical analysis.

## Results

**Fig. 3.**
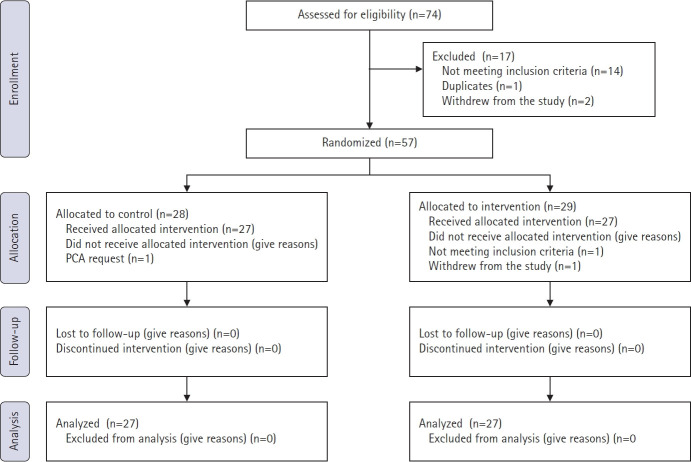
Study flow diagram. PCA, patient-controlled analgesia.

A total of 74 patients were assessed for eligibility, of whom 17 were excluded, including 14 who did not meet the inclusion criteria, one duplicate case, and two who withdrew from the study. Therefore, 57 patients were randomized and allocated to either the control group (n=28) or the intervention group (n=29). Of the 57 randomized patients, three were excluded during the study: one from the control group owing to the need for additional pain control, one from the intervention group for not meeting the inclusion criteria, and one who withdrew. Consequently, the final analysis included 27 patients in each group (total n=54) (Fig. 3). The baseline characteristics and perioperative data of both groups showed no significant differences and were similar with respect to age, sex, body mass index, and ASA physical status (Table 1). There were no reports of adverse events related to the QL block. There were no significant differences in the duration of surgery or anesthesia between the two groups. The QL group had a significantly shorter PACU stay of 10 minutes (median, 20 minutes [IQR, 20–25] vs. 30 minutes [IQR, 25–40]; *p*<0.001). Both groups had similar intraoperative fluid requirements, remifentanil dosages, and incision lengths. The QoR-15 scores at 24 hours postoperatively were also similar between the two groups. No significant differences were observed between the groups in terms of the first flatus time, postoperative complications, length of hospital stay, or readmission rates.

**Fig. 4.**
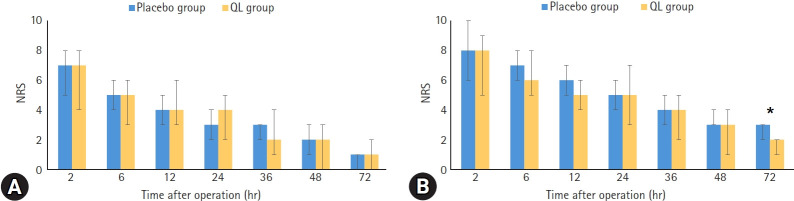
Numeric rating scale pain score for (A) pain at rest and (B) movement-induced pain. The bar graph shows the median, and the error bar shows the interquartile range. NRS, numeric rating scale; QL, quadratus lumborum. *p=0.01.

The NRS scores for pain at rest and movement-induced pain at each time point are shown in Fig. 4. The primary outcome, NRS pain score at rest at postoperative 6 hours, showed no statistically significant difference between the placebo and QL groups (*p*=0.78). There were also no significant differences in the pain at rest NRS scores from 2 to 72 hours postoperatively. The QL group had significantly lower NRS scores for movement-induced pain at postoperative 72 hours than the placebo group (median, 2 hours [IQR, 1–2] vs. 3 hours [IQR, 2–3]; *p*=0.01). However, the pain scores were similar between the groups at other time points. Until 72 hours postoperatively, both groups required similar doses of additional tramadol and showed no significant differences. The number of patients in the QL group who received pethidine between 2 and 6 hours postoperatively was lower than that in the placebo group (8 [29.6%] vs. 2 [7.4%]; *p*=0.03) (Table 2). Total analgesic use was monitored from immediately after surgery to 72 hours postoperatively. Morphine equivalents were calculated using an opioid equianalgesic chart, with tramadol converted at a ratio of 1 to 0.1 mg morphine and pethidine at 1 to 0.13 mg morphine [21,22]. Although less analgesic was administered to the QL group, the difference was not statistically significant (mean±standard deviation, 3.3±1.3 mg vs. 2.5±1.1 mg; *p*=0.39). The PONV symptoms were similar in both groups at all time intervals. Four patients in the placebo group received antiemetics during 2 to 6 hours and 6 to 12 hours postoperatively, whereas no patients in the QL group required antiemetic treatment (*p*=0.03) (Table 2).

## Discussion

Although several studies have reported the beneficial effects of abdominal wall blocks, their efficacy remains controversial [23,24]. Several clinical trials have indicated the potential of QL blocks to facilitate and improve postoperative pain management in patients undergoing laparoscopic colorectal surgery [25,26]. Moreover, QL blocks have been shown to provide longer duration of analgesia and reduce the need for additional analgesics than TAP blocks [23,27], which supports the QL block as one of the principal components of multimodal analgesia protocols. However, some studies have indicated that opioid consumption and pain scores were not positively affected by QL block [24,28-30] compared to placebo. The results of the present study support those of recent publications highlighting the limitations of QL blocks in abdominal surgeries. This double-blind, randomized, placebo-controlled trial demonstrated the absence of a significant difference in patient postoperative NRS pain scores after a preoperative unilateral QL block following ileostomy closure. Furthermore, while the number of patients requiring rescue pethidine at 2 to 6 hours postoperatively and the movement-induced NRS pain scores at 72 hours postoperatively differed between the two groups, the total analgesic use and NRS pain scores at other time points did not differ.

Several reasons have been attributed to the lack of benefits of the QL block [24,30]. One of the reasons for the limited effectiveness of abdominal blocks [30] is thought to be visceral pain, which remains largely unaffected by regional blocks and continues to be most effectively treated with systemic opioids. During the design of this study protocol, visceral irritation was assumed to be limited during ileostomy closure; and the primary source of postoperative pain was considered to be somatic pain resulting from the single incision on the right lower abdomen. Therefore, based on patient-reported symptoms, a unilateral QL block was expected to be effective in controlling postoperative pain. A recent study evaluated the efficacy of QL blocks for stoma closures [31]. In that study, stoma surgeries (creation, revision, and closure) were performed on 137 patients (84%). Accordingly, the consumption of opioids was limited to 0.7 oral morphine milligram equivalents/kg on the first postoperative day, and the pain score was less than 2 over 3 postoperative days following the QL block. However, the participants in that study were children and a control group was not included for comparison purposes. In contrast, our study included patients who had previously undergone stoma creation. Central sensitization related to the initial surgery and the presence of a stoma may have contributed to postoperative pain, distinguishing this cohort from the de novo surgical cases. Central sensitization refers to a state in which neurons in the central nervous system become more excitable so that even normally non-painful stimuli can be perceived as painful (allodynia) and painful stimuli are felt more strongly than usual (hyperalgesia). This altered processing amplifies pain signals and renders patients less responsive to standard analgesic treatments. In this situation, regional techniques, such as the QL block, may not be sufficient to control visceral or centrally mediated pain. This may explain why pain relief was less noticeable in our cohort, even when the block was performed correctly. Therefore, our results indicate that central sensitization should be recognized as an important factor when evaluating the limited effectiveness of QL blocks in patients with prior abdominal surgery and stoma creation [32].

There is a possibility of technical failure or insufficient infiltration of local anesthetic. A single expert anesthesiologist with >10 years of experience performed the QL block under ultrasound guidance; however, postoperative sensory block was not directly assessed. A European study previously indicated that sensory loss after an anterior QL block in laparoscopic colorectal surgery was unpredictable in terms of patient outcomes [24]. Although the QL group had more patients with sensory blocks than the placebo group, opioid use and pain scores were similar. Another study on laparoscopic ovarian tumor resection found that the QL block covered more dermatomes and reached higher cephalad levels than the TAP block [33]. However, similar to other fascial plane blocks, the QL block has an inconsistent sensory spread [34] and requires further investigation.

The sample size was calculated based on patient-reported NRS pain scores. The primary outcome was the NRS pain score at 6 hours postoperatively, with opioid consumption as the secondary outcome. The 6-hour time point was chosen because it aligns with the known efficacy duration of a single QL block, and by this time, the patients are expected to be fully awake and available for interviews. However, given that the terminal half-life of ropivacaine is <6 hours post-surgery, the effect of the QL block could diminish by this time. This might explain the lower frequency of rescue pethidine use in the QL group during the first 2 to 6 hours postoperatively in the present study. The NRS pain score is subjective and opioid consumption is considered the primary outcome in most clinical trials because reduced opioid use benefits patient recovery. Supplemental opioid use can also depend on subjective symptoms and patient preferences, emphasizing the need for objective pain-related outcome measures for postoperative pain management.

The QL group had significantly fewer patients requiring rescue antiemetics than the placebo group, despite no significant differences in PONV symptom scores. However, this result did not lead to improved overall recovery outcomes and should be interpreted with caution owing to the limited sample size. The absence of a difference in QoR-15 scores between the groups further supports this finding, suggesting that the reduction in rescue antiemetic use did not translate into a measurable improvement in the overall recovery quality.

Our study had several limitations. First, at 2 hours post-surgery, the NRS pain scores were as high as 7 at rest and 8 during coughing in both groups, indicating insufficient pain management. This may be attributed to the limited visceral coverage of the QL block, which primarily targets somatic pain in the abdominal wall, and the short duration of its effect due to the single-shot injection technique. In addition, the previous multimodal regimen lacked adjunctive wound infiltration and optimized systemic analgesics, potentially reducing overall analgesic synergy. We modified the pain management protocol to provide more intensive management for ileostomy closure. The adapted protocol includes wound infiltration (with either a single injection or continuous infiltration of ropivacaine), or use of Welpass (Genewel Co., Ltd.) and administration of postoperative nefopam and celecoxib. Second, the extended study period is a limitation of this study. The overlap with the coronavirus disease-2019 pandemic led to delays in patient recruitment and prolonged the overall study duration. Although the standardized protocol and outcome assessments were consistently maintained, we considered the possibility of a hidden bias arising from the study’s extension, as hospital policies and clinical practices may have changed during that period. Third, because QL blocks were performed under general anesthesia, technical success prior to surgery could not be assessed. Fourth, the relatively small sample size may have limited the statistical power of the study and restricted the interpretation of the results.

In our randomized controlled trial, the ultrasound-guided QL block did not demonstrate a significant advantage over placebo in terms of postoperative pain control in patients undergoing ileostomy closure. Based on these findings, routine use of QL blocks in ileostomy closure appears to offer no substantial benefit. Future studies are warranted to determine the optimal concentration and volume of local anesthetics for ileostomy closure, and to clarify whether the QL block may provide benefits in specific patient subgroups.

## Figures and Tables

**Table 1.** Basic characteristics and perioperative outcome

Characteristic	Placebo group	QL group	*p*-value
No. of patients	27	27	
Age (yr)	58.5±10.5	59.0±10.8	0.75
Sex			0.75
Male	20 (74.1)	21 (77.8)	
Female	7 (25.9)	6 (22.2)	
Body mass index (kg/m^2^)	23.3±2.9	22.7±2.9	0.75
ASA PS classification			>0.99
I^a)^	2 (7.4)	2 (7.4)	
II^b)^	25 (92.6)	25 (92.6)	
Intraoperative fluid (mL)	457.4±200.8	518.5±194.7	0.86
Intraoperative remifentanil (µg)	387.0±73.8	313.6±99.5	0.73
Duration of operation (min)	56.2±17.9	60.0±14.9	0.44
Duration of anesthesia (min)	79.8±20.4	87.9±18.9	0.49
PACU time (min)	30 (25–40)	20 (20–25)	<0.001
Incision length (cm)	4.0 (4–5)	4.0 (4–5)	0.30
First flatus time (hour)	46.1±18.3	39.7±20.4	0.98
QoR-15, score	114.5±13.4	115±17.1	0.13
Hospital stay (day)	5 (4, 5)	5 (4, 6)	0.91
Postoperative complication	1 (3.7)	2 (7.4)	0.24
Ileus	0 (0)	2 (7.4)	
Voiding difficulty	1 (3.7)	0 (0)	
Readmission	0 (0)	0 (0)	-

Values are number only, mean±standard deviation, number (%), or median (interquartile range). QL, quadratus lumborum; ASA, American Society of Anesthesiology; PS, physical status; PACU, post-anesthesia care unit; QoR-15, Quality of Recovery-15.

a) Normal healthy patient,

b) patient with mild systemic disease.

**Table 2.** Rescue drug use and incidence of PONV

Variable	Postoperative time (hr)	Placebo group (n=27)	QL group (n=27)	*p*-value
Tramadol	0–2	5 (18.5)	4 (14.8)	0.71
	3–6	3 (11.1)	2 (7.4)	0.63
	7–12	2 (7.4)	4 (14.8)	0.38
	13–24	3 (11.1)	3 (11.1)	>0.99
	25–48	4 (14.8)	1 (3.7)	0.15
	49–72	4 (14.8)	1 (3.7)	0.15
Pethidine	0–2	2 (7.4)	5 (18.5)	0.22
	3–6	8 (29.6)	2 (7.4)	0.03
	7–12	2 (7.4)	1 (3.7)	0.55
	13–24	2 (7.4)	3 (11.1)	0.63
	25–48	2 (7.4)	1 (3.7)	0.55
	49–72	0 (0)	0 (0)	-
Metoclopramide	0–2	1 (3.7)	1 (3.7)	>0.99
	3–6	4 (14.8)	0 (0)	0.03
	7–12	4 (14.8)	0 (0)	0.03
	13–24	1 (3.7)	0 (0)	0.31
	25–48	2 (7.4)	3 (0.0)	0.63
	49–72	0 (0)	0 (0)	-
Ramosetron HCl	0–2	0 (0)	0 (0)	-
	3–6	1 (3.7)	0 (0)	0.31
	7–12	0 (0)	0 (0)	-
	13–24	1 (3.7)	1 (3.7)	>0.99
	25–48	0 (0)	0 (0)	-
	49–72	0 (0)	0 (0)	-
PONV	0–2	6 (22.2)	4 (14.8)	0.84
	3–6	5 (18.5)	2 (7.4)	0.50
	7–12	5 (18.5)	2 (7.4)	0.42
	13–24	1 (3.7)	2 (7.4)	0.61
	25–36	1 (3.7)	2 (7.4)	>0.99
	37–48	0 (0)	2 (7.4)	0.23
	49–72	0 (0)	0 (0)	-
Total analgesic use (mg)	0–72	3.3±1.3	2.5±1.1	0.39

Values are range or mean±standard deviation. PONV, postoperative nausea and vomiting; QL, quadratus lumborum; HCl, hydrochloride.
